# Reconceptualising Behavioral and Psychological Symptoms of Dementia: Views of People Living With Dementia and Families/Care Partners

**DOI:** 10.3389/fpsyt.2021.710703

**Published:** 2021-08-16

**Authors:** Claire V. Burley, Anne-Nicole Casey, Lynn Chenoweth, Henry Brodaty

**Affiliations:** ^1^School of Psychiatry, Dementia Centre for Research Collaboration, University of New South Wales (UNSW) Sydney, Sydney, NSW, Australia; ^2^School of Psychiatry, Centre for Healthy Brain Ageing, University of New South Wales (UNSW) Sydney, Sydney, NSW, Australia; ^3^Academic Department for Old Age Psychiatry, Prince of Wales Hospital, South Eastern Sydney Local Health District, Sydney, NSW, Australia

**Keywords:** BPSD, changed behavior, neuropsychiatric symptoms, lived experience, agitation, anxiety, depression, person-centered care

## Abstract

**Background:** Behavioral and psychological symptoms of dementia (BPSD, also known as neuropsychiatric symptoms (NPS), changed behaviors and responsive behaviors), occur in up to 90 percent of people living with dementia (PLWD). These symptoms and behaviors strongly correlate with functional and cognitive impairment and contribute to ~30% of overall dementia costs. As decisions regarding care and strategies for BPSD are generally based on professional frames of reference, this study investigates whether the perspectives of PLWD and families/care partner on BPSD terminology can inform a more nuanced conceptualization of BPSD.

**Methods:** PLWD and families/care partners participated in one-on-one semi-structured interviews. A thematic iterative approach was used to code the data and identify common themes until theoretical saturation was reached. Themes were compared between groups. Data were analyzed deductively in relation to pre-existing terminology regarding BPSD, and inductively to discover new ideas on use of such terminology as perceived by PLWD and others.

**Results:** Forty-one volunteers were interviewed: 21 PLWD, mean age 71 yrs, mean Mini-Mental State Examination score 25, and 20 family members/care partners. Three main themes emerged from the data: (1) descriptions of BPSD from people with lived experience compared to clinical terms, (2) viewpoints on interpreting causes, and (3) experiences of concurrent BPSD. The experiences described and terms used by PLWD and families/care partners differed from terms used in existing professional frameworks (e.g., “disinhibition” described as ‘loss of filter') and there were differences between PLWD and family members' interpretations of BPSD causes.

**Discussion/Conclusion:** Reports from PLWD and families/carers describing their experiences of BPSD suggest a reconceptualization of BPSD terminology is needed to understand and de-stigmatize these symptoms and behaviors. For example, the term “agitated/hard to handle” would benefit by clearer, contextualized description, such as “frustrated with cognitive decline, discriminatory behavior and inadequate support systems.” In better understanding individual expressions of BPSD, families, professionals and societies will be able to respond in ways that are helpful for PLWD. An informed, integrated understanding of BPSD and improved terminology use will have the potential to improve the quality of care and support for PLWD.

## Introduction

Behavioral and psychological symptoms of dementia (BPSD; also known as neuropsychiatric symptoms (NPS), changed behaviors and responsive behaviors) are estimated to affect approximately 90 percent of people living with dementia (PLWD). BPSD strongly correlate with functional and cognitive impairment (1). BPSD terminology includes aggression, agitation, anxiety, apathy, depression, disinhibited behaviors, nocturnal disruption, psychotic symptoms, vocally disruptive behaviors and wandering (2). These symptoms and behaviors can cause distress for PLWD (3, 4), families and/or care partners (5) and care staff (6), and they impose a financial burden on society in relation to the cost of informal and formal support services, contributing approximately 30 percent of overall costs (7–9).

### BPSD Framework: Historical and Current Context

The operational definition of BPSD was developed following an international consensus conference organized by the International Psychogeriatric Association (IPA) in 1996 (2), after recognizing that the term “challenging behaviors” was viewed as prejudicial by many consumers. The IPA group noted that many persons with dementia experience symptoms of depression, psychosis and several other BPSD that elicited far less clinical attention, and research support, compared to cognitive symptoms. Since then, BPSD has become widely used to describe over 10 different behaviors and psychological symptoms that may be experienced by PLWD. BPSD nomenclature is used as a framework for measurement in clinical practice and research, and has informed clinical practice guidelines.

While evidence-based clinical guidelines provide extensive information and advice to assist clinicians and informal carers with BPSD issues (6, 10, 11), gaps remain in understanding the experience of BPSD from the perspective of PLWD (12).

The limitations of using BPSD terminology to describe a complex range of changed behaviors and psychological symptoms has been identified by clinicians, informal carers and PLWD. It has been suggested that specific attention be given to describing individual BPSD (e.g., depression, anxiety, sleep disturbances) using language that reflects the person's experience (13). The language used to describe BPSD is important to question, because such terminology has the power to negatively influence thoughts, beliefs, emotions and behavior toward PLWD (14, 15). Cunningham et al. (13) caution, however, that regardless of attempts to reduce stigma by adopting an alternative terminology to BPSD, common usage of alternative terminology eventually becomes pejorative and stigmatizing in its own right. Deconstruction of BPSD terminology indicates that further research could help in better understanding behavioral and neuropsychiatric symptoms as experienced by PLWD (12) and close family (14).

Few studies have directly sought the views of PLWD on their experiences and interpretation of BPSD, the way that they handle these experiences and what they consider to be suitable terminology to describe the specific BPSD they experience. One study investigated the views of people with mild to moderate dementia toward BPSD terminology “wandering” (16). The authors concluded that a reconceptualization of wandering behavior terminology was required, suggesting that labeling wandering as aimless walking and physical disruption did not reflect the purposeful and beneficial activity of wayfinding and/or exploring one's environment. Thus, reconceptualization of BPSD terminology that more accurately describes the person's experiences and their interpretation of them, will likely have dramatic effects on the way that families/informal carers and clinicians respond to PLWD; this may influence the person's self-perception and care practices (16). Ultimately, obtaining information from those with direct experience of BPSD can inform current BPSD guidelines and enhance support systems.

### Aims and Hypotheses

We aimed to identify and compare the experiences, interpretation and approaches to BPSD for PLWD (PWLD) and their families/care partners, and to obtain their views on terminology describing BPSD. We hypothesized that PLWD would have different views and use different language to describe their experiences of BPSD when compared to families/care partners and terms used in existing medical frameworks, for example, terms used in the Neuro-Psychiatric Inventory (17). The findings from this study may guide future research in this area and inform clinical guidelines, practice, research and improve care.

## Materials and Methods

### Study Design

In this prospective qualitative study, we conducted semi-structured interviews with PLWD and their family members and/or care partners. The study received ethical approval from the South Eastern Sydney Local Health District (SESLHD) Research Strategy Office (RSO) and the University of New South Wales (UNSW) Sydney Human Research Ethics Committee (HREC), HREC: 2019/ETH09814. The study methods adhered to the Standard for Reporting Qualitative Research [(18); see Supplementary Material 1].

### Participant Recruitment and Setting

Where possible, we recruited participant dyads—the PLWD and a close family member/care partner (e.g., a person and their spouse). Most of the participants were recruited at arms-length through the online platform StepUp for Dementia Research (https://www.stepupfordementiaresearch.org.au/). Study posters and flyers providing details of inclusion criteria were also displayed in SESLHD outpatient areas (i.e., waiting rooms) and on memory clinic notice boards, inviting people meeting the inclusion criteria to take part in the research. Study flyers were also distributed electronically through social media platforms (Dementia Centre for Research Collaboration (DCRC), StepUp for Dementia Research and Centre for Healthy Brain Ageing (CHeBA) websites and Twitter). There were no pre-existing relationships between participants and researchers except for one personal contact who was given the first author's contact details (CB) by the last author (HB). This participant was sent the participant information sheet and consent form (PISCF) and communicated in the same way as other participants.

Prior to screening, potential participants were given a PISCF detailing the names, backgrounds and contact details of the researchers, information about the study aims and procedures, and what participation would involve. Screening involved checking eligibility for participation either face-to-face at UNSW Sydney, over Zoom or by phone. A safety protocol was established as part of the study protocol to protect the well-being of participants.

For PLWD, inclusion criteria included persons of any age with a formal diagnosis of dementia (any subtype), willing and able to give informed consent and able to participate and comply with the study protocol. Exclusion criteria for PLWD were inability to give informed consent and/or inability to demonstrate understanding of what study participation involves, and lead researcher (CB)-assessed Mini-Mental State Examination [MMSE; (19)] score of <18 out of 30. Inclusion criteria for family members/care partners were that they must be willing and able to give informed consent and must know the PLWD well (i.e., either living with them or see them ≥4 h per week).

Following screening and consent, participants who met eligibility criteria were invited to take part in an interview of between 45 and 90 min. Participants met the lead researcher either at UNSW Sydney (prior to the COVID-19 pandemic), online via Zoom or Skype, or by telephone, as preferred. The lead researcher asked participants if they were willing for their responses to be audio-recorded. If unwilling, they were advised that they could still participate, and that the researcher would instead hand-record their responses to interview questions (i.e., field notes). Participants were asked if they would like to receive the study findings. Those who expressed interest were contacted at the end of the study and provided a summary of the results. They were then invited to provide feedback.

A pilot of the interview procedure was performed with six participants (3 PLWD, 3 family members/care partners). Following reflection on interview procedures, adjustments to the interview schedule and approach were made to interview style and format of questioning. These amendments helped to ensure that the format of questioning was clear and sensitive, whilst allowing flexibility in questions to suit the interviewee. For example, some people required more prompting, while others found talking about particular issues difficult, so the lead researcher adapted each interview accordingly. Participant recruitment and data collection continued until data saturation was reached (20).

### Date Collection

Interviews were conducted by the lead project investigator (CB, PhD). During interviews, questions asked included describing behaviors identified in the BPSD terminology that participants either experienced or observed. These questions made reference to BPSD sub-categories formed *a priori* to interviews, for example, agitation, depression, delusions, but were couched using terminology that was possibly more likely to be experienced, for example, frustration, seeing or hearing things [see (6)]. Participants were asked to advise on helpful and unhelpful approaches in responding to the different behaviors that were experienced and/or observed, and on preferred terminology for describing BPSD. In recognizing the potential of not capturing all participant behaviors and symptoms experienced and/or observed in referencing questions to the BPSD framework, we included the following open-ended questions at the beginning of the interview, “*Have you noticed any changes in your behavior?”* and at the end of the interview*, “Are there any other changes in your behavior that you have experienced that we haven't already talked about'?”* Participants were also invited to give advice on changes needed to improve support for PLWD and where research efforts should be focused. Questions were posed to all participants in a sensitive manner to avoid any labeling or negative connotation (i.e., by presenting possible scenarios and avoiding terms such as “BPSD”).

### Data Entry and Storage

Interviews were transcribed verbatim by lead author CB who then checked transcript accuracy by listening to and cross checking the transcriptions with the audio-recordings. Names of people and locations in the recording were removed from the transcribed data, to ensure participant confidentiality. Participants were allocated unique identifier codes (e.g., 01A). Electronic copies of the audio-recordings and interview transcripts were stored in a password-protected computer system on the secure University server. Only the researchers had access to the secure electronic files. Transcribed data were not returned to participants for comment or correction because this was not considered necessary in addressing the aims of this research.

### Date Analysis

We used a hybrid approach of thematic analysis that incorporated both the deductive *a priori* template of codes approach to more focused research questions (21) (see Supplementary Material 2), and the data-driven inductive approach for the open-ended question responses (22). This complementary approach to data analysis supported our research aims by allowing existing BPSD terminology (e.g., a particular behavior/symptom) to be integral to the process of deductive thematic analysis, while allowing for themes to emerge directly from the data using inductive coding. Themes were developed from the agreed data codes for all questions, ceasing only when no new themes emerged from the data, that is, until data saturation was reached (23–25).

Data familiarization was conducted independently by authors CB and AC, who read through the interview transcripts to get an overview of the depth, richness and diversity of the data collected. Transcribed interview data were inspected independently by CB and AC, who tagged participant responses (excerpts) that directly corresponded to the *a priori* interview questions and coded the transcripts line-by-line. The relevant data codes and supporting excerpts were managed and organized on Microsoft Word and tabulated under each research question and compared. A third researcher (LC) then reviewed the allocated codes alongside the supporting quotes. The research team members held multiple meetings to discuss, compare, and document their independent and shared findings through an iterative process. Any discrepancies in findings were discussed until consensus was achieved on the final codes that emerged from the data. The common themes arising from the agreed codes were identified and named by CB, AC and LC through close inspection and discussion of the participant excerpts associated with the agreed data codes (26).

Responses between PLWD and families/care partners were also compared. Due to the small size and diversity of the sample, agreement between responses and other findings are described narratively without the use of descriptive statistics.

### Safety and Risk Considerations

Participants could choose to end their participation in the study at any time. The interviewer closely monitored participants throughout to ensure minimal distress, including signs of fatigue and/or confusion in persons with dementia. There was a potential for such risks, given the nature of the topics being discussed. The interviewer was sensitive to these risks and frequently reassured participants during the interview, emphasizing they did not have to answer any questions they may have felt uncomfortable with. If the participant showed any signs of distress the researcher paused interview questions and asked the participant whether they would like a break or to end the interview. Participants were also offered contact details and resources for relevant support services and invited to contact the researcher if they had any concerns regarding the study. One participant who was a PLWD experienced mild distress during the interview and chose to end their interview. The researcher expressed gratitude for their involvement and time and assured them that their data would not be used and that it was completely acceptable for them to not want to continue. Another participant who was a carer became tearful during the interview, though insisted they wanted to continue. The researcher supported them throughout, offered regular breaks and contact details for support services, and sent a follow-up email to ensure they were ok. Family members/care partners found the process of being involved in the interview helpful for their own needs, which they felt they had been neglected due to the demands of being a carer.

## Results

The results reported in this article include responses from questions related to specific experiences of BPSD (e.g., frustration, nervousness) and any other changes in behavior participants experienced or observed (see Supplementary Material 2, responses to part (a) of all numbered questions are presented here). The themes presented relate to language used to describe experiences and interpreting causes, from PLWD and families/care partners. Detailed analysis on helpful and unhelpful responses (see Supplementary Material 2, responses to part (b) of all numbered questions) and views toward umbrella terms (e.g., “BPSD”, “responsive behaviors”, “changed behaviors”) will be the subject of forthcoming publications.

### Participant Characteristics

Forty-one participants were recruited and interviewed between December 2019 and February 2021. Recruitment was temporarily paused due to physical distancing requirements initiated during the COVID pandemic (March–July 2020). Interviews then recommenced online only. Participants were 21 PLWD and 20 family members/care partners. Of the 21 PLWD, one chose to withdraw during the interview and one participant's data were lost due to a corrupt recording file. The remaining 39 participants included 14 dyads (PLWD and their family member/care partner). Participants were interviewed on their own except for three PLWD who completed their interview in the presence of their family member as a support person. The family member/care partner was then interviewed separately. Participant demographic characteristics are summarized in Tables 1, 2. Table 2 provides additional information regarding dementia subtype and severity, other medical conditions, other difficulties (e.g., mobility, communication) and medications.

**Table 1:** Participant characteristics.

	**Person living with dementia (*n* = 21)**		**Family members/care partners (*n* = 20)**
Sex	Female (14)	**Sex**	Female (14)
	Male (7)		Male (6)
Age	70 ± 7.39	**Relationship to person living with dementia**	Partner (13)
			Daughter/son (5)
			Carer (1)
			Friend (1)
Place of residence	Private with family (17)
	Private alone (3)
	Residential care (1)
Australian state of residence	NSW (10)	
	VIC (5)	
	QLD (4)	
	WA (2)	

*NSW, New South Wales; QLD, Queensland; VIC, Victoria; WA, Western Australia*.

**Table 2:** Additional characteristics of people living with dementia.

Dementia type	AD (5)	Other medical conditions	>1 other condition (10)
	FTD (4)		None (8)
	LBD (1)		Mood disorders (10)
	Mixed/other (11)		Sleep disorders (9)
			High BP (6)
			Cancer (4)
			Other (6)
Dementia severity	Mild (12)	Other difficulties	>1 other difficulty (3)
	Moderate (6)		None (11)
	Unknown (3)		Mobility (6)
			Communication (4)
			Hearing (3)
			Intellectual (1)
			Unknown (2)
MMSE score	25 ± 4.55	Medications	>1 medication (7)
			None (3)
			Dementia (8)
			Mood disorders (6)
			Other (11)
			Unknown (5)

*AD, Alzheimer's disease; BP, blood pressure; FTD, frontotemporal dementia; LBD, lewy body dementia; MMSE, mini-mental state examination. “Other” medical conditions included cardiac problems, diabetes, or stroke. Other medications included treatment for cancer, stroke, gastrointestinal conditions, pain, high cholesterol and high blood pressure*.

### Main Findings

The analysis resulted in 622 data codes and three main themes from responses to interview questions about any changes in behavior participants may have experienced or observed (depending on whether the participant was a PLWD or family/care partner) and about specific individual BPSD (e.g., frustration, nervousness; see Supplementary Material 2). The findings presented below are structured around the main themes relating to the aims of this article.

**Figure 1:**
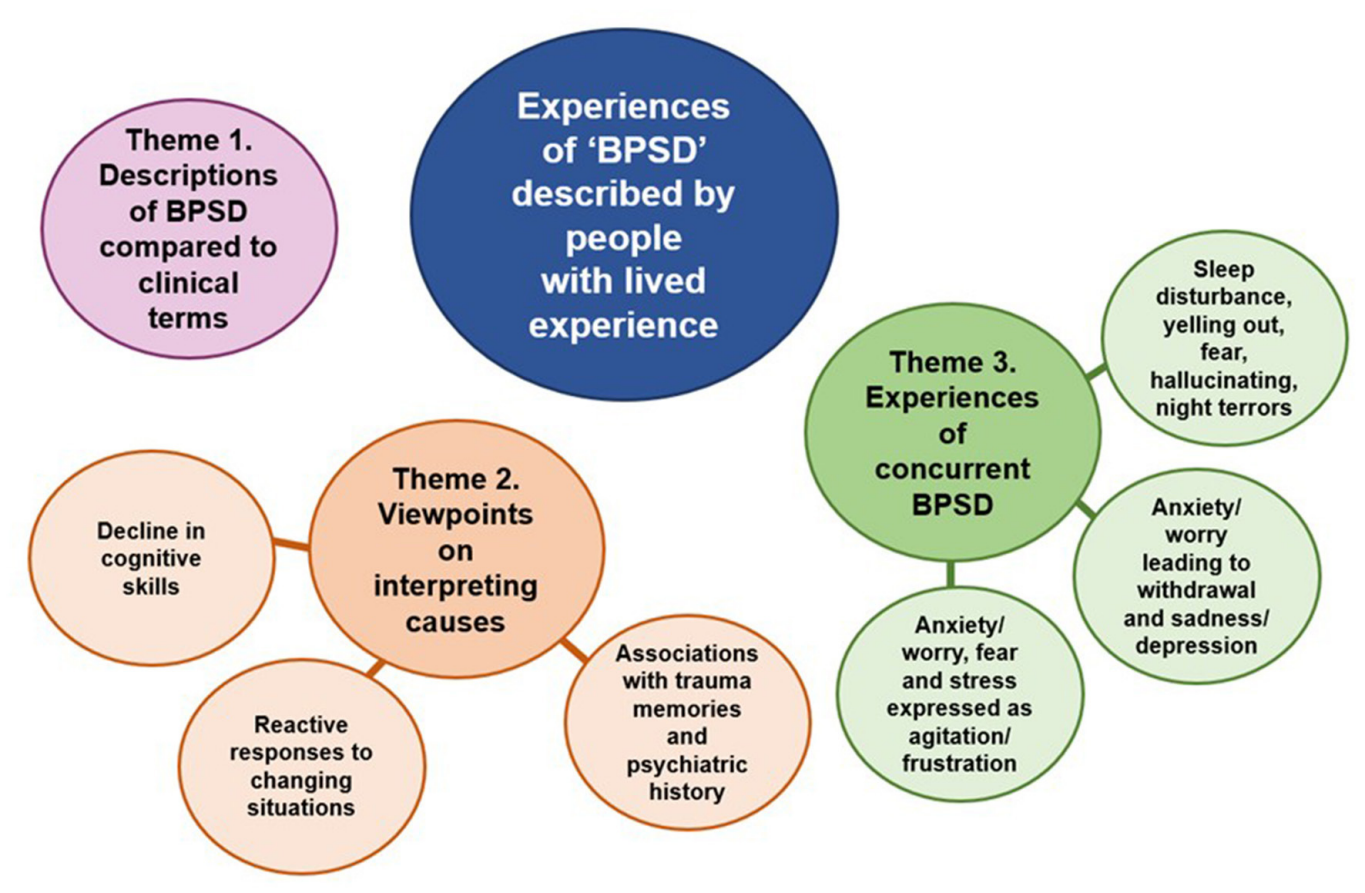
Summary of main themes and subthemes that emerged relating to the aims of this article.

We utilized the opportunity to incorporate views of PLWD and families/care partners by presenting all the findings from the interviews together. Quotations from PLWD are marked with a “P,” family members/care partners with a “C,” and matching numbers show where quotations came from a dyad (e.g., P07 and C07 are the person and their spouse). An overview of three main themes and their subthemes is shown in Figure 1. There were variations in the contributions of different perspectives to each of the themes where theme 1 and 2 received similar contributions from both perspectives, whereas theme 3 was driven more by the perspectives of PLWD.

### Theme 1. Experiences of BPSD Described by People With Lived Experience Compared to Clinical Terms

**Figure 2:**
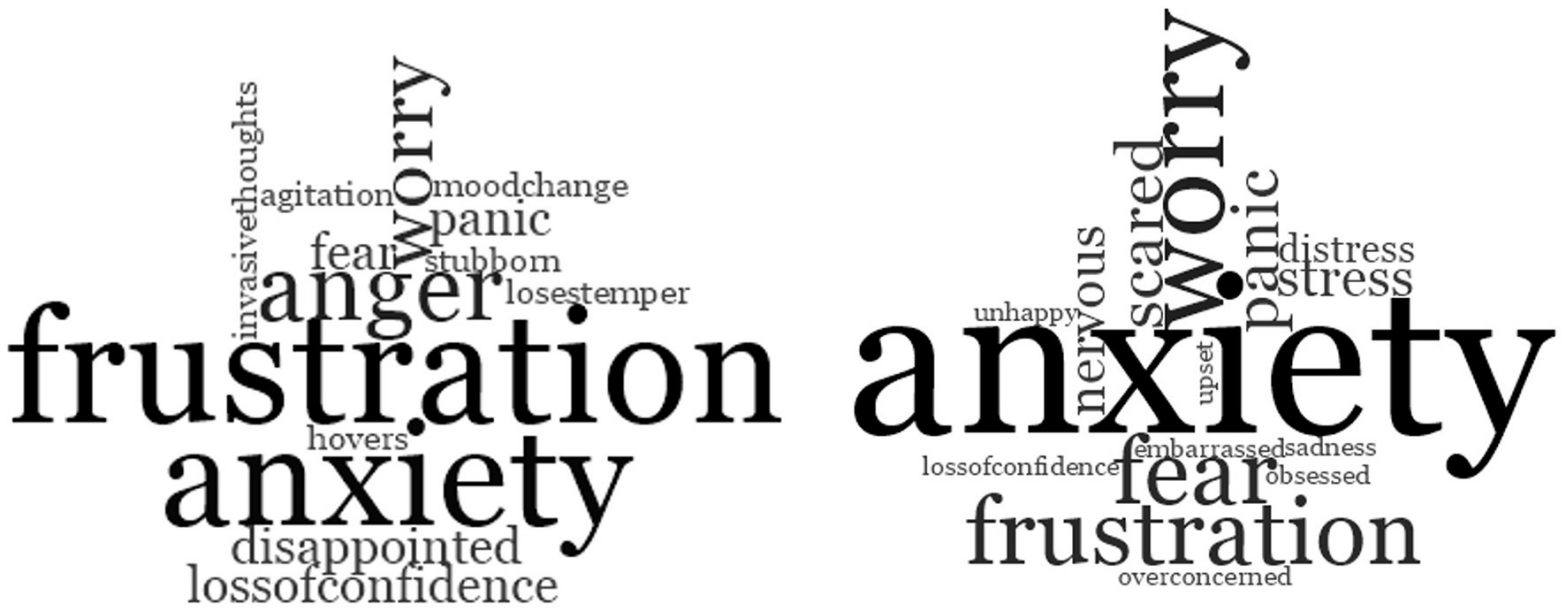
Word clouds showing frequency of word use when participants were describing **(left)**. experiences focused on “agitation,” and **(right)** experiences focused on “anxiety”.

PLWD and families/care partners use alternative terms to clinical terms used in professional frameworks when describing their experiences (see Table 3). Clinical terms include agitation, aggression, anxiety, apathy, depression, disinhibited behaviors, sleep disturbance, psychotic symptoms (delusions and hallucinations), vocally disruptive behaviors, wandering and aberrant motor behaviors (17). PLWD and families/care partners' use of alternative terms varied considerably and there was overlap between individual BPSD (see theme 3). Table 3 details alternative terminology use and example quotes. Figure 2 shows frequency of alternative terms used when describing experiences related to agitation and anxiety.

**Table 3:** Terminology^*^ used to describe individual behavioral and psychological symptoms of dementia (BPSD) by people with lived experience.

**Clinical term (deductive approach)**	**Alternative terms (inductive approach)**	**Example quotes**
Aggression	Raging, angry, anger, lashing out, contempt, confusion, frustration, insecurity, self-loathing, fear	P02: *I have a right to be **angry**. The way I'm expressing my anger now is different to how I would have done things in the past*. P04: *I get **angry** that I can't remember, and I know I should be able to remember*. C21: *That was sort of, strange because I've never seen him, he would never lash out. And it was just frustration from him. But he'd never done anything like that before*. P25: *I don't want to turn into this nasty woman. I'm feeling **aggressive**, which I never did before*.
Agitation	Frustration, intolerance, impatience, annoyance, fear, stress, worry, panic, anxiety, loss of confidence, overwhelmed, empathize	P01: *It's more **frustrating** when it (involves) someone close to you because **you**feel their hurt*. C02: *She gets **frustrated** with doctors and professionals not believing that she, you know, has all these symptoms because she functioned quite well*. C02: *She gets very **frustrated** with people saying that everyone forgets, ‘oh that happened to me the other day'. It's just sort of brushed off. ‘Oh, well, it's just part of aging'*. P05: *You get **frustrated** when people just don't know how to treat you. One minute they're treating you as if there's nothing left up top and the next minute, they're treating you as if there's no problems*.
Anxiety	Scary, worry, nervous, tentative, frightened, fear, sadness, loss, upset, withdraw, loss of independence	P26: *Having dementia is really **scary**. Every day, you don't quite know where you are. You don't know who's with you. You don't know if you're at home. You don't know if you're safe …you've lost everything that makes you, you*. P02: *Things that I'm **frightened** of are things that could happen, based on reality so it's hard to take away*. P04: *I'm **worried** I'm thinking how long is he going for?* *I just need to know where he is, what he is doing*. *(her husband)* P12: *I have a lot of **anxiety**. I wanna stop feeling that way. I don't like it because I'm **scared**, it's like a **fear**, it's like a fear and you just think I'll go home and I then I don't have to deal with anyone*. C12: *She'll get **anxiety** about going to the shops or going to these programs*.
Apathy/ indifference	Lacking motivation, lacking interest, lacking energy, isolated, withdrawal, disengagement, cognitive overload, dependency on carer/loss of independence, memory loss, communication difficulties, loss of confidence, content, busy	P18: I've had so much to do I haven't been able to do it lately so I've just let it slip (walking). I'm fairly contented with the way my days and weeks operate. C18: *He's now much more **withdrawn**. We're becoming more and more **isolated** from each other because it's the only way that I can survive in this relationship*. P12: *I go inside myself. I'm avoiding people now. I can't hold a conversation. I'm lost in my head. I'm going around in circles to keep my mind on track*. C12*: They could have had a busy week; they're just not feeling up to it. (They) might have an appointment tomorrow that (they're) worried about and just want to relax at home*.
Depression	Sad, low, hurt, emotional, muted, down, pessimistic, grief, emotional crying mess, upset, feels loss, distressed, grief, rumination, shock, vulnerable, flat, withdrawn, fear, inward looking, anxious, anger, insular, frustration, lucky, relief	P11: *I hadn't planned for that in my life. That shouldn't have happened. I was too young*. C11: *There's been a sense of loss I suppose which is only natural, I guess*. C17: *She used to get very sad about losing her, she lost her half-brother in the Second World War. We get stuck in sad memories sometimes*. P18: *I've had very strong feelings of sadness, about erm I to my wife and how she reacts or vice versa, I find it very difficult*. C18: *He doesn't share. He will occasionally make reference to the fact he has a problem. We are now so distant*. P19: *The people you love can't stand you, you can't stand yourself. What's the point? I wanted to die*. C19*: She's been through this **grief**. (The) fixation on death and fears that she was displaying seemed more like a **depression***.
Disinhibited behavior	Loss of filter, upset people, blunt speech, impulsive, outspoken, embarrassment, thinking out loud, demanding, outraged, inappropriate, provocative, frivolous, loss of insight, intolerance, irritation,maintains tight control	P01: *Tolerance to some things is much less. I couldn't cope with the attitudes of the staff*. P02: *I swear more than I used to swear. I've never been somebody that swears much, but I seem to do it more now*. P04: *He (spouse) puts it politely, ‘Your filter is going'. I don't really remember. I believe I'm getting worse, because a couple of times he's been, ‘Do you realize what you just said?' ‘No what did I say?' ‘Oh, did I?' I can't even remember*.
		P12: *I have no filter. I just come out and say it. It's just what I really think. Yea and that's when I get into arguments with everyone*. C12: *Sometimes she feels that people don't understand her at all, and she really does verbally try and get her point across, she's very outspoken*.
Sleep disturbances	Go to sleep earlier, wake up earlier, wake in the middle of the night, unable to get to sleep, sleeplessness, sleep longer, fatigue, slump, nap, tired in the daytime, irritation, tiredness, bad dreams, crazy dreams, sleep walking, Sundowners syndrome, nervous, scared, increased sensitivity, frustration, isolated, lonely	C01*: (They are) sleeping all day and waking all night. And then at night, when she needs people around or she needs some comforting, there's no one there. So, she's actually probably feeling quite isolated and lonely. So, I can only just imagine how horrible it must be*. C13: *[He] doesn't sleep as well as he used to, to be honest, but he sleeps better during the day than he does at night*. P25: *I might go to sleep for an hour or two hours and then I'm awake for two or three hours and yea my sleeping pattern is horrible*. P25: *Being in residential care, you've got someone yelling out and then it wakes you. I couldn't go back to sleep and I was just going back to sleep and they yelled out again, so I was awake again*.
Psychosis (delusions/hallucinations)	Night terrors, yelling in sleep, imagining, altered perception, discrimination, dreaming, wonders why people are helping, conspiring, colluding, defensive, hurtful, distressing, paranoia, misreading social cues, nervous, misidentification, high sensitivity, assuming, false beliefs, scared, aggressive, misreading situations	P02: *I don't necessarily see things clearly. So, shadows, sometimes they're a bit distorted. I think sometimes I see movement that probably isn't there. It's just a light reflection or something. But I wouldn't classify them as hallucinations*. P03*: I've been chased by wild animals. Things like that. There are people trying to get into the house at night. That sort of thing. Just at night-time when I'm sleeping*. C03: *(They) went through a long stage of getting out of bed and banging against the walls and trying to fight off the bears*. C07: *(They) think that we conspire, we collude. (They) get very defensive. We try and explain, (they) just think the secrets are against (them)*. P07: *I thought I was talking with somebody. I was actually talking to the mirror*. *Which is quite embarrassing when you're in a store and everybody can see this nutcase talking to a mirror*.
Vocally disruptive behavior	Calling out, memory lapses, repeating things, humming, anxiety, agitation, fear, shouts out for help	C01: *They're **calling out** for somebody who you can't get, they've passed away*. C08: *(They) ask the same question half a dozen times or more. Just repeating over and over* C12: *(They do) repeat things, tell you something and then the next five minutes, say it over and over*. P11: *I called for help. And I thought someone was trying to drag me off. I thought I was going to be raped or whatever*.
Wandering/aberrant motor behavior	Frequent/excessive walking, purpose, exercise, walking	P02: *I think all the walking's for a purpose. We walk for a purpose. Our purpose may not be very clear. I know I walk around more now*. P02: *So, I'm walking more than I realize, but it's often, cause I'm going somewhere and then I don't know why I've gone there. So, I go and do something else. I get easily distracted. So, I end up wandering around looking, working out what I was doing*. P02: *We're all searching for what we can't remember we're searching for*. C16: *It's quite normal to want to go outside for a walk, it's just they can't verbally express they want to*. C17: *She liked to walk, and she would head out to go, not necessary to realistic places. Quite often it was going right back to her childhood home and she was determined. So, it wasn't aimless, it just wasn't realistic*.

* *Bold text shows words with similar meanings to clinical terminology. Underlined text shows words with different meanings to clinical terms, highlighting overlap between feelings/symptoms. Double-underlined text shows where people are expressing behaviors/feelings that oppose clinical terms. Numbers indicate dyads (e.g., P12 is PLWD and C12 is their carer)*.

### Theme 2. Viewpoints on Interpreting Causes of BPSD

This theme outlines the main similarities and differences in viewpoints on interpreting causes of BPSD perceived by PLWD and families/care partners. It has three subthemes: decline in cognitive skills, reactive responses to changing situations, and associations with trauma memories and psychiatric history. Summaries are provided with example quotations.

#### Subthemes: Decline in Cognitive Skills and Reactive Responses to Changing Situations

Both PLWD and families/care partners interpreted BPSD as being reactive (i.e., as responses to changing circumstances), or resulting from deterioration in the person's cognitive skills. Families/care partners more frequently interpreted BPSD as resulting from a decline in cognition and self-determination, thus, affecting their abilities to respond in ways and live life as they had previously. BPSD were also associated with increased thinking about traumatic memories by PLWD and their families/care partners.

*Some frustration in not being able to do things that have been easier. And also, some frustration because you're doing something that you've previously found to be, in effect a no thought involved exercise, and despite being very careful about trying to make sure you do it correctly you do make, some fundamental errors (P01)*.

*I do get sad with myself when I wish I'd remember these things (P04)*.

*She does get frustrated because she can't remember (C04)*.

*(PLWD) gets frustrated with doctors and professionals not believing that (they), you know, has all these symptoms because (they) function quite well (C02)*.

When comparing responses from dyads, sometimes the PLWD and their family/carer would provide conflicting explanations as to why a particular behavior was expressed. In the example below, the PLWD assigns self-isolating behavior as a way of avoiding confrontation because they feel they are not accepted socially. In contrast, the family member/care partner describes them as losing interest and motivation. This may parallel what occurs with clinicians, where a behavior is observed to have occurred but the motive behind the behavior is not understood.

*I've got to stay by myself and don't bother anyone. If people can't understand who I am and except me. Because we've all got to accept everyone for their bad faults. And I go inside myself. I'm avoiding people now I suppose. I can't hold a conversation. I'm lost in my head. I'm going around in circles to keep my mind on track and that I'm answering the question right (P12)*.

*I noticed with one of our programs (they) used to really like going to the activities. (They) haven't had interest over the past few weeks. Just getting up sometimes and having the shower can be really hard (C12)*.

The data indicate that the feelings PLWD express about changed behavior are plausible responses to the situations they find themselves in and/or their interpretation of the situation. The family carers, on the other hand, tended to limit their interpretation to observed changes in the person's expressed feelings and responses to certain situations. PLWD rarely mentioned apathy, agitation, wandering, vocally disruptive behaviors or psychological symptoms, but did mention anxiety, sleep disturbance and disinhibition. Family members/care partners were more likely to mention agitation, anxiety, apathy, sleep disturbance, psychological symptoms and some mentioned disinhibition. Most PLWD and carers/family members acknowledged aggression, but their responses to anger/aggression terminology varied. Some carers and PLWD may have downplayed depressive symptoms, but both carers and PLWD acknowledged sadness in response to the impact of dementia on their lives. Both PLWD and carers discussed the impact of others' responses in various contexts and what was/wasn't helpful.

#### Subtheme: Associations With Trauma Memory and Psychiatric History

Feeling unsafe and fearful was also common. Both PLWD and families/care partners identified that persistent memories of previous traumatic events contributed to difficult emotions such that PLWD “*would get stuck in painful memories.”* Some participants may have been experiencing post-traumatic stress disorder symptoms that were further compounded with memory difficulties and increased rumination and focus on previous traumatic events (e.g., serving on the frontline during the war, previous physical and sexual abuse, difficult family histories and dynamics, abduction). History of mental illness was also a contributing factor in some PLWD, sometimes making it difficult (and possibly inappropriate) to disentangle experiences caused by dementia-related brain changes and pre-existing psychological symptoms.

*There are just so many traumatic things that have happened. She's definitely much more emotional, more focused on that, and it will spin off every time she is watching TV or talking to people. There will always be some sort of a reference to one of those things (C16)*.

*She used to get very sad about losing her half-brother in the Second World War. We get stuck in sad memories sometimes (C17)*.

*She's got depression and she's got bipolar, and she's had it off and on all that time* (34 years) *but as I said with the illness that she's got now* (dementia) *it's gradually getting worse than as I said a few years ago. It's probably a little bit more frequent than what it has been (C24)*.

### Theme 3. Experiences of Concurrent BPSD

Rather than describing the experience of distinct behaviors and psychological symptoms, most people interviewed described multiple concurrent feelings and behaviors within the same experience (e.g., anxiety/worry expressed as agitation/frustration; anxiety leading to withdrawal, presenting as apathy, then leading to depression; see Table 3 and Figure 2). The subthemes identified include “anxiety/worry, fear and stress experienced as agitation/frustration,” “anxiety/worry leading to withdrawal and sadness/depression,” and “sleep disturbance, yelling out, fear, hallucinations and night terrors” These are described in more detail with relevant quotations below.

#### Subtheme: Anxiety/Worry, Fear and Stress Expressed as Agitation/Frustration

When describing experiences related to agitation, common experiences included “*frustration*,” “*intolerance*,” “*impatience*,” “*fear*,” “*stress*,” “*worry*,” “*panic*,” “*anxiety*,” “*annoyance*” and “*loss of confidence*.” Often, several terms were used to describe one experience. In summary, reasons for feeling anxiety, frustration, worry, fear and panic described by PLWD and family members/carers included difficulties with thinking, feeling unsupported, feeling unsafe and fearful, and losing independence and sense of identity. There was considerable overlap between terms used to describe experiences related to “anxiety” and “agitation” (Figure 2). Difficulties with thinking included not remembering things, being slower at doing things, remembering things unclearly, skill loss and impaired cognition, reasoning and understanding.

*I've had other situations where the environment has become very stressful, and I've had to really escape from. I suppose the best description would be like an anxiety attack. Just the chaos of (people protesting) coming out onto the street and cheering, became very disorientating. I just had to really run from there and get away. It was sort of conflicting in that, it was something I would have really supported, would have loved to have been involved with. The environment, it was overpowering. The number of people, the noise. And just the difficulty of trying to move through a crowd. You're really trapped in that environment. It's very hard to get out (P01)*.

Many participants felt unsupported in the community and in health care settings. In general, they considered that others in the community did not know how to treat PLWD. Some PLWD felt that they wanted “*somewhere in-between”*: to be treated as they had been prior to receiving a diagnosis, but also with acceptance and understanding of the unique difficulties that they may be experiencing.

*You get frustrated when people just don't know how to treat you. One minute they're treating you as if there's nothing left up top and the next minute, they're treating you as if as if there's no problems. And somewhere in between is actually better. That gets very frustrating (P05)*.

PLWD and families/care partners expressed inconsistent experiences of professional support. Some felt “*very lucky”* not to be experiencing a particular behavior (e.g., depression, disinhibition) or to have the healthcare professionals involved with their care, whereas others felt completely unsupported or that the quality of professional support they received was inadequate and were left feeling disappointed.

*I came away disappointed because she* (clinician) *didn't bother looking at my film* (brain images/scans). *She just read the report and went off that. But I wanted her to make up her own mind. I wanted a second opinion. I went away frustrated. I paid all this money for nothing (P12)*.

PLWD expressed frustration and/or sadness over lost independence (e.g., no longer being able to drive), “grief” over lost sense of self, and “letting go” of plans they had for the future that had been replaced by fear of the future. In some cases, these feelings were further exacerbated by feelings of “guilt” over the impact of these losses on their partner and a need to reassess their lives.

*I'm sad sometimes that I'm not giving [partner] the life that you know (they) should have. It makes me sad sometimes when you know when (they're) stuck here with me and not able to do things (P21)*.

*(They were) just really fearful about the future and very fixated on death and dying. (They) had a number of really key friends and relationships that ended through horrible deaths (C19)*.

*(Their) mum had dementia, they worry about going down a similar path (C24)*.

*It's a grief process. The whole idea of the life that you thought that you're going to have together has been thrown off kilter. So, you've really got to give that up. You've got to let it go and find a different way. And that can be really hard (C23)*.

#### Subtheme: Anxiety/Worry Leading to Withdrawal and Sadness/Depression

Some PLWD expressed not wanting others to know about their diagnosis for fear of how they would be treated. They would often try to hide their diagnosis and as this became increasingly difficult, they would withdraw from others and reduce the number of social activities they engaged in. Some participants described feelings of sadness and experiencing depression as a result. Other family members/care partners described the person withdrawing as an expression of worrying about remembering people. However, PLWD expressed varying responses with regard to the role of independence on their ability to socialize and take part in activities. Some described needing a carer to help motivate them, whereas others described the ongoing presence of a carer as inhibiting their ability to socialize.

*I find it harder that (spouse) is there all the time because I'm not having this interaction (with) others. I'm very sociable and I've always been very sociable (P04)*.

*I know what worries her is that she can't remember people. She withdraws all of it. It's making those connections (C04)*.

*I like to have (spouse) close by. If not him then another carer. In case I forget something or whatever. I'm dependent on someone else taking me wherever we go (P11)*.

*It's good to have a carer here because she gets me out and she motivates (me). It can be a bit difficult. My motivation has gone downhill (P12)*.

*I'm scared and I feel I need to withdraw because I don't want to upset people (P25)*.

Other common experiences included lacking interest, loss of energy, cognitive overload, non-stimulating activity program, and not identifying with others who have dementia.

#### Subtheme: Sleep Disturbance, Yelling Out, Fear, Hallucinations, Night Terrors

Sleep disturbance was reported less frequently than other BPSD with some sleep changes being associated with yelling out, hallucinations and night terrors, sometimes linked to traumatic memories (theme 2, subtheme).

*I've been chased by wild animals. Things like that. There are people trying to get into the house at night. That sort of thing. Just at night-time when I'm sleeping (P03)*.

*(They) went through a long stage of getting out of bed and banging against the walls and trying to fight off the bears. He yells (C03)*.

*The nightmares the dream nightmares I was having were so real, they were so real I had to get out of bed, to walk around to wake up enough to, you know almost pinch almost hurt myself, to know that that's a dream it's not real (P19)*.

## Discussion

The main aims of this research were to investigate the experiences of BPSD and terminology used by PLWD, to explore possible reasons for their experiences, and to summarize the main similarities and differences between the views of PLWD and the views of family/care partners. The main themes were divergent terminology use in describing experiences by PLWD and families/care partners compared to clinical terms used by health professionals (theme 1, Table 3), interpretation and attribution of BPSD from PLWD and families/care partners (theme 2), and experiences of concurrent BPSD (theme 3, Figure 2). PLWD were more likely to attribute experiences as reactive responses to changing situations whereas families/care partners were more likely to explain behaviors resulting from a decline in cognitive skills. Factors such as relationship type, living arrangements, age of onset and dementia type may have influenced what/how participants chose to discuss and acknowledge, and the levels of agreement between PLWD and families/care partners. Previous traumatic experiences and psychiatric history were often discussed when interpreting causes. While BPSD are currently considered under large umbrella terms (e.g., BPSD) or as separate behaviors/psychological symptoms (agitation, anxiety, depression, etc.) themes 1, 2 and particularly theme 3 suggested that several individual BPSD are often intrinsically linked within an experience (e.g., agitation perceived by others is an expression of anxiety felt by PLWD caused by fear about the future).

Our findings of the subjective experience of behaviors and symptoms of PLWD are similar to those described by others (3, 14, 16). In their examination focused on PLWD with Lewy Bodies, Larsson et al. (3) summarized similar themes around disease impact, in terms of symptom experience, restricted participation and activities, and self-perception. Van Wijngaarden and colleagues' study explained how PLWD are affected by “the eyes of others” and longed for a safe and accepting environment, but quite often felt scrutinized by inquisitive and disapproving looks (4). The authors described how much of the person's suffering stems from living under the shadow of negative imaginaries [i.e., a broad understanding of the way a given people imagine their collective social life; (27)], similar to experiences that emerged in our subtheme of “reactive responses to changing situations.” Exploration of loss and identity changes experienced by PLWD, regarding relationships with their own body, others and the surrounding world (4), echo subthemes of decline in cognitive skills and loss of identity and confidence that were found in our research. Our study further identified the contribution of historical trauma and psychiatric history, encapsulating the role of short-term memory loss and disorientation where long-term memory and historic views of self are given increased prominence in current experiences.

Adekoya and Guse's (16) study, which focused on the behavioral term “wandering,” described themes emerging from language used by older adults with mild to moderate dementia and suggested a reconceptualization of wandering behavior, from aimless walking and disruption to a purposeful and beneficial activity (16). Similarly, Gilmore-Bykovskyi et al. (14) found considerable language use variability between families/care partner and clinicians and highlighted the importance of evaluating language in partnership with PLWD, whilst responding to carers' interpretations of BPSD and sense-making. Our findings that the descriptions provided by people with lived experience and carers do not fully support the application of BPSD terminology commonly used by clinicians and researchers align with other reports (14, 16).

### Implementing Person Centered Care and the Influence of Language

The themes “*Viewpoints on interpreting causes”* and “*Experiences of concurrent BPSD”* highlight the common finding that there is no universal experience of dementia (28). These findings contribute to those of previous studies (3, 14, 16) that suggest a need for health professionals to consider these personal experiences and interpretations in refining terminology, which provides the basis for more individualized and person-centered responses. Previous studies have shown that person-centered approaches are effective in improving well-being and life experiences for PLWD (29, 30), however there are challenges in implementing person-centered care at scale (31).

In our study PLWD described plausible feelings about and responses to losing cognitive abilities and loss of control. Concurrently, clinical psychological/psychiatric symptoms do occur, and it is important that these are not neglected (6, 17). BPSD terminology regarding the person's needs and decisions around care used in frameworks and guides must be accurate, up to date, and most importantly, in agreement with those with lived experience of dementia. Ensuring a mutual understanding between professionals and people with lived experience will help ensure the most appropriate and effective treatment or care approach is used.

### Strengths and Limitations

A major strength of this study is that we obtained descriptions of experiences and views from people with lived experience of dementia, either as someone living with a diagnosis, or someone who is a close family member, carer of friend. Participants had varying types of dementia diagnoses and severity, many had comorbidities and other health conditions and were taking several medications (see Table 2), making it difficult to determine whether experiences described are solely due to their dementia diagnosis or other factors. All the participants volunteered to take part in this research study and findings may not be generalizable to all PLWD or to larger populations, as those who volunteer in research studies may have different motivations, values and beliefs that may also influence the experience of BPSD. Other factors that may influence BPSD include unmet needs, environmental factors (e.g., lack of activity, safety issues) and carer factors (e.g., stress and communication difficulties).

The knowledge and engagement of the researchers (some had both professional and personal experience with dementia) in talking, listening to and interpreting information provided by PLWD and families/care partners can be considered a strength, because of their in-depth experience and background knowledge. However, this could also be regarded as a limitation as it may make the researchers biased in their interpretations of the results. The analytical approach of two researchers independently coding data, a third researcher reviewing the data, and the discussions about differing interpretations aimed to reduce this potential bias.

### Implications and Conclusion

Few research studies seek the views of PLWD themselves about the changes that they experience. The views of people with lived experience of dementia, particularly those with a diagnosis, are vital in better understanding their experiences and reasons that they interpret as contributing to them. This information then needs to be shared appropriately with all relevant stakeholders who play a part in influencing the landscape of dementia care more broadly. It is anticipated that our findings will inform clinical guidelines and practice and improve treatment and care options for PLWD, with a particular focus on knowledge translation. The findings also lay the foundation for a new area of research focusing on an integrated approach that investigates the views of PLWD, families/care partners as well as healthcare professionals, providers, government workers and policy makers on BPSD. An integrated understanding of all the views involved will assist in improving language use and the appropriateness, quality and availability of care and support.

## Data Availability Statement

The raw data supporting the conclusions of this article will be made available by the authors, without undue reservation.

## Ethics Statement

The studies involving human participants were reviewed and approved by South Eastern Sydney Local Health District (SESLHD) Research Strategy Office (RSO) and UNSW Sydney Human Research Ethics Committee (HREC), HREC: 2019/ETH09814. The patients/participants provided their written informed consent to participate in this study.

## Author Contributions

CB designed the study, collected data, carried out and transcribed interviews, analyzed the data, and wrote the article. A-NC assisted with data analysis and writing the article. LC gave expert guidance on analysis, assisted with data analysis and writing the article. HB designed the study, supervised data collection and analysis, and assisted with writing the article. All authors contributed to the article and approved the submitted version.

## Conflict of Interest

The authors declare that the research was conducted in the absence of any commercial or financial relationships that could be construed as a potential conflict of interest.

## Publisher's Note

All claims expressed in this article are solely those of the authors and do not necessarily represent those of their affiliated organizations, or those of the publisher, the editors and the reviewers. Any product that may be evaluated in this article, or claim that may be made by its manufacturer, is not guaranteed or endorsed by the publisher.
