# Supplementary material for: Reconceptualising Behavioral and Psychological Symptoms of Dementia: Views of People Living With Dementia and Families/Care Partners
Source: Front Psychiatry. 2021 Aug 16;12:710703. doi: 10.3389/fpsyt.2021.710703 (PMC8415310; doi:10.3389/fpsyt.2021.710703)
Supplement: Supplementary file 2 [file Data_Sheet_2.DOCX]

## SUPPLEMENTARY MATERIAL

### **S1. COREQ (COnsolidated criteria for REporting Qualitative research) Checklist (O’Brien et al. 2014)**

Please see attached completed COREQ form and additional detail below.

COREQ Item 4 & 5:

The interviewer (lead investigator CB) was a female qualified research psychologist with clinical experience of working with PLWD, their family/carers and health professionals.

COREQ Item 7:

All participants received information about the authors prior to the study on the Participant Information Sheet and Consent Form (PISCF). The authors and researchers of this study all have a particular interest in improving care for PLWD, through better understanding the changes they experience and promoting research activities that are inclusive of PLWD.

COREQ Item 17:

See Supplementary Material S2. Questions used in interview schedule for PLWD and families/ care partners.

### **S2. Questions used in interview schedule for PLWD and families/ care partners**

Responses to questions 1 - 12 part (a) are presented in this paper. Responses to questions 1 – 12 part (b) and questions 13 – 16 will be the subject of forthcoming papers.

*Please note: This schedule is to be used flexibly as guidance and should be adapted to suit the individual being interviewed. Prompt questions only need to be used if person does not elaborate on experience or naturally talk about what they find helpful/ unhelpful.

In-between each question, check participant is ok and offer a break if any doubt.

*I’m going to ask you about any changes in behaviour that you/ (PLWD’s name) may have experienced since [you were/ (PLWD’s name was)] diagnosed with dementia. I would like you to talk to me about these changes, how they made you feel and things that you found helpful or unhelpful. There are no right or wrong answers as we really want to better understand your experience.*

[clinical term: BPSD]

1. [general open-ended question]
   1. *Have you noticed any changes in your/ (PLWD’s name) behaviour since being diagnosed with dementia?*
   2. *What have you found helpful/ unhelpful when you experienced (use participant’s wording)? What would you like to happen? What do you think should happen?*

[clinical term: agitation]

1. *Sometimes people get frustrated more easily than they did before. These feelings may be directed towards themselves. They may be more likely to occur in certain situations.*
   1. *Have you/ (PLWD’s name) experienced this or something similar?*
   2. *What have you found helpful/ unhelpful when you experienced (use participant’s wording)? What would you like to happen? What do you think should happen?*

[clinical term: aggression]

1. *Some people can feel very angry and sometimes lash out at people they love. This maybe because of miscommunication in addition to confusion caused by the thinking and memory difficulty.*
   1. *Have you/ (PLWD’s name) experienced this or something similar?*
   2. *What have you found helpful/ unhelpful when you experienced (use participant’s wording)? What would you like to happen? What do you think should happen?*

[clinical term: anxiety]

1. *Some people become very nervous. For example, they may find tasks difficult that they used to find easy, such as their morning routine of getting up, getting dressed and having breakfast. Sometimes people show other signs of nervousness such as shortness of breath, being unable to relax, or feeling very tense.*
   1. *Have you/ (PLWD’s name) experienced this or something similar?*
   2. *What have you found helpful/ unhelpful when you experienced (use participant’s wording)? What would you like to happen? What do you think should happen?*

[clinical term: apathy]

1. *Sometimes people lose interest in things they previously enjoyed. This may be because some activities become more difficult due to the memory and thinking difficulties.*
   1. *Have you/ (PLWD’s name) experienced this or something similar?*
   2. *What have you found helpful/ unhelpful when you experienced (use participant’s wording)? What would you like to happen? What do you think should happen?*

[clinical term: depression]

1. *It is common for people to feel very sad and perhaps experience more sadness than usual.*
   1. *Have you/ (PLWD’s name) experienced this or something similar?*
   2. *What have you found helpful/ unhelpful when you experienced (use participant’s wording)? What would you like to happen? What do you think should happen?*

[clinical term: *disinhibited behaviors]*

1. *Sometimes people do or say things that they wouldn’t do normally. For example, some people may say things that might upset others, that they would normally keep to themselves. Some people undress in public and others may say sexually inappropriate things or touch others inappropriately.*
   1. *Have you/ (PLWD’s name) experienced this or something similar?*
   2. *What have you found helpful/ unhelpful when you experienced (use participant’s wording)? What would you like to happen? What do you think should happen?*

[clinical term: nocturnal disruption]

1. *It is common for people to experience problems sleeping, for example: difficulty getting to sleep, waking during the night, rising too early or taking many naps during the day.*
   1. *Have you/ (PLWD’s name) experienced this or something similar?*
   2. *What have you found helpful/ unhelpful when you experienced (use participant’s wording)? What would you like to happen? What do you think should happen?*

[clinical term: delusions]

1. *Some people might start thinking that others are stealing from them or trying to harm them in some way when this isn’t true.*
   1. *Have you/ (PLWD’s name) experienced this or something similar?*
   2. *What have you found helpful/ unhelpful when you experienced (use participant’s wording)? What would you like to happen? What do you think should happen?*

[clinical term: hallucinations]

1. *Some people may hear voices or see things that aren’t really there.*
   1. *Have you/ (PLWD’s name) experienced this or something similar?*
   2. *What have you found helpful/ unhelpful when you experienced (use participant’s wording)? What would you like to happen? What do you think should happen?*

[clinical term: vocal disruptive behaviour]

1. *Sometimes people start making noise that can cause stress to others in their environment. For example, they may start screaming, shouting or being verbally abusive, or they may start making more subtle sounds such as groaning or sighing.*
   1. *Have you/ (PLWD’s name) experienced this or something similar?*
   2. *What have you found helpful/ unhelpful when you experienced (use participant’s wording)? What would you like to happen? What do you think should happen?*

[clinical term: wandering]

1. *Some people experience a strong need to keep moving and will want to walk or do other things repeatedly (for example fiddling with objects, handling buttons or wrapping string).*
   1. *Have you/ (PLWD’s name) experienced this or something similar?*
   2. *What have you found helpful/ unhelpful when you experienced (use participant’s wording)? What would you like to happen? What do you think should happen?*

[terminology focused questions]

*I’m going to ask you about different terms that are used to describe these behaviors. I will give you an example then ask you if you’ve heard of the term and how it makes you feel (whether you agree/ disagree/ feel neutral).*

1. *Behavioural and psychological symptoms of dementia (BPSD)*
   1. *Have you heard of this term before?*
   2. *How does this term make you feel?*
2. *Responsive behaviors*
   1. *Have you heard of this term before?*
   2. *How does this term make you feel?*
3. *Changed behaviors*
   1. *Have you heard of this term before?*
   2. *How does this term make you feel?*
4. *Unmet needs*
   1. *Have you heard of this term before?*
   2. *How does this term make you feel?*
5. *Thinking back to all the things we have talked about, is there anything else that you would like to share?*

*Many thanks for your time*
